# Urinary complement proteins and risk of end-stage renal disease: quantitative urinary proteomics in patients with type 2 diabetes and biopsy-proven diabetic nephropathy

**DOI:** 10.1007/s40618-021-01596-3

**Published:** 2021-05-27

**Authors:** L. Zhao, Y. Zhang, F. Liu, H. Yang, Y. Zhong, Y. Wang, S. Li, Q. Su, L. Tang, L. Bai, H. Ren, Y. Zou, S. Wang, S. Zheng, H. Xu, L. Li, J. Zhang, Z. Chai, M. E. Cooper, N. Tong

**Affiliations:** 1 Division of Nephrology, Laboratory of Diabetic Kidney Disease, Centre of Diabetes and Metabolism Research, West China Hospital of Sichuan University, No. 37, Guoxue Alley, Chengdu, 610041 Sichuan Province China; 2 Division of General Practice, West China Hospital of Sichuan University, Chengdu, Sichuan China; 3 Laboratory of Diabetic Kidney Disease, Centre of Diabetes and Metabolism Research, West China Hospital of Sichuan University, Chengdu, Sichuan China; 4 Key Laboratory of Transplant Engineering and Immunology, MOH, West China Hospital of Sichuan University, No. 37, Guoxue Alley, Chengdu, 610041 Sichuan Province China; 5 West China-Washington Mitochondria and Metabolism Research Center, West China Hospital of Sichuan University, No. 37, Guoxue Alley, Chengdu, 610041 Sichuan Province China; 6 Frontiers Science Center for Disease-Related Molecular Network, West China Hospital of Sichuan University, No. 37, Guoxue Alley, Chengdu, 610041 Sichuan Province China; 7 Histology and Imaging Platform, Core Facility of West China Hospital, Chengdu, Sichuan China; 8 Division of Pathology, West China Hospital of Sichuan University, Chengdu, Sichuan China; 9 Department of Diabetes, Central Clinical School, Monash University, Melbourne, Australia; 10 Division of Endocrinology, West China Hospital of Sichuan University, Chengdu, Sichuan China

**Keywords:** Complement, Urinary proteomics, Diabetic nephropathy, End-stage renal disease

## Abstract

**Purpose:**

To investigate the association between urinary complement proteins and renal outcome in biopsy-proven diabetic nephropathy (DN).

**Methods:**

Untargeted proteomic and Kyoto Encyclopedia of Genes and Genomes (KEGG) functional analyses and targeted proteomic analysis using parallel reaction-monitoring (PRM)-mass spectrometry was performed to determine the abundance of urinary complement proteins in healthy controls, type 2 diabetes mellitus (T2DM) patients, and patients with T2DM and biopsy-proven DN. The abundance of each urinary complement protein was individually included in Cox proportional hazards models for predicting progression to end-stage renal disease (ESRD).

**Results:**

Untargeted proteomic and functional analysis using the KEGG showed that differentially expressed urinary proteins were primarily associated with the complement and coagulation cascades. Subsequent urinary complement proteins quantification using PRM showed that urinary abundances of C3, C9, and complement factor H (CFAH) correlated negatively with annual estimated glomerular filtration rate (eGFR) decline, while urinary abundances of C5, decay-accelerating factor (DAF), and CD59 correlated positively with annual rate of eGFR decline. Furthermore, higher urinary abundance of CFAH and lower urinary abundance of DAF were independently associated with greater risk of progression to ESRD. Urinary abundance of CFAH and DAF had a larger area under the curve (AUC) than that of eGFR, proteinuria, or any pathological parameter. Moreover, the model that included CFAH or DAF had a larger AUC than that with only clinical or pathological parameters.

**Conclusion:**

Urinary abundance of complement proteins was significantly associated with ESRD in patients with T2DM and biopsy-proven DN, indicating that therapeutically targeting the complement pathway may alleviate progression of DN.

**Supplementary Information:**

The online version contains supplementary material available at 10.1007/s40618-021-01596-3.

## Introduction

The global pandemic of diabetes mellitus (DM) was reported to have reached approximately 463 million adults in 2019 [1]. Diabetic nephropathy (DN), develops in approximately 21.3% of patients with DM [2], and has become the leading cause of end-stage renal disease (ESRD) worldwide. The individual and societal costs associated with treating DN are very high. The median cost of inpatients with chronic kidney disease (CKD) and DM was US $2288 and US $2102 per person per year, respectively, according to the CKD Network 2015 Annual Data Report [3]. Identifying risk factors for progression to ESRD in patients with DM is crucial to reduce morbidity, mortality, and the social and economic impact of DN burden. Historically, the onset and progression of kidney damage in diabetes widely relied on clinical parameters, such as estimated glomerular filtration rate (eGFR) and albuminuria [4]. However, recent evidence suggests that these parameters have limited value. A high frequency of remission of microalbuminuria or regression of macroalbuminuria to microalbuminuria was observed in type 2 diabetes mellitus (T2DM) [5, 6]. In the setting of T2DM, the need to identify novel prognostic biomarkers and therapeutic targets for progression in DN is urgent.

The pathogenesis of DN has traditionally been viewed to involve renal hemodynamic changes, oxidative stress, hypoxia, overactivation of the renin–angiotensin–aldosterone system (RAAS), and modification of molecules under hyperglycemic conditions (i.e., advanced glycation end-products) [7]. Inflammation also plays crucial roles in the development of DN, as evidenced by the infiltration of immune cells observed in glomeruli and interstitium of renal biopsy samples at all stages of DN [8]. Increasing evidence indicates that the complement system plays a pivotal role in the onset and progression of renal disease, including DN [8]. Complement proteins are deposited in the kidneys of patients with DN. Increased tubular complement component 5 (C5) deposition and glomerular C4 were detected in patients with DN, and the intensity of C5 or C4 staining was strongly associated with kidney disease progression in these patients [9, 10]. Complement activation has been implicated in regulation of renal tubulointerstitial injury in patients with DM and proteinuric CKD. Notably, experimental models of diabetes revealed that blocking C3a/C3aR or C5a/C5aR signaling reduced tubulointerstitial fibrosis and alleviated inflammation of the injured kidney [10, 11]. These studies confirmed a causal link between complement proteins and progression of DN under conditions of T2DM.

Urine is a fluid produced by the kidneys that can provide information on renal pathophysiology. Moreover, urine is easily sampled noninvasively, and urinary proteins are stable and resistant to sudden degradation [12]. Owing to the development of proteomic analysis of kidney diseases, many proteomic analyses of urine from diabetic patients were conducted to identify urinary biomarkers that can predict the progression of nephropathy [13] and early decline in eGFR [14]. Notably, in a Mexican–American cohort of 141 patients with T2DM and proteinuric CKD, quantification of urinary levels of 12 complement proteins using parallel reaction-monitoring (PRM) liquid chromatography–mass spectrometry (LC–MS) revealed urinary abundance of complement C4 and C8, and complement regulatory proteins CD59 and factor H-related protein 2 (FHR2) were strongly associated with progression to ESRD and all-cause death. However, natural history and clinicopathological features of DN may differ between patients of different ethnicity. A study of biopsied Chinese patients with diabetes found that the prevalence of non-diabetic renal disease (NDRD) was 35% [15]. Furthermore, urinary proteomic biomarkers differed significantly between DN and NDRD [16]. Few studies considered the association between the severity of renal pathology and complement proteins. Therefore, studies that analyze the association between urinary complement proteins and renal histological changes as well as that between complement proteins and ESRD in patients with biopsy-proven pure DN are needed. In the present study, we investigated the association between urinary complement proteins and progression to ESRD in patients with T2DM and biopsy-proven pure DN. The association between urinary complement proteins and renal histological changes was aslo evaluated.

## Materials and methods

### Patient selection and study design

Diabetic patients who underwent renal biopsy from 2010 to 2018 at West China Hospital of Sichuan University were screened. Indications for renal biopsy were diabetes and renal damage with persistent albuminuria or renal dysfunction, particularly in those with sudden onset overt proteinuria or hematuria [15]. Criteria of the American Diabetes Association were used to diagnose T2DM [17]. DN was defined according to the standard reported by An et al. [18] in 2015 and was diagnosed by at least two renal pathologists and/or nephrologists based on the Renal Pathology Society (RPS) classification [19]. To accurately characterize trajectories of eGFR decline, we restricted our analysis to the following patients according to our previous study [20]: (1) those in whom serum creatinine levels were measured at least three times per year during the follow-up period; and (2) all serum creatinine assays were performed at our hospital to minimize methodological differences. Therefore, the exclusion criteria were: (1) coexisting non-diabetic kidney disease and systemic diseases, especially those involving antineutrophil cytoplasmic antibodies, such as vasculitis, antiglomerular basement membrane disease, and lupus nephritis; (2) nontype 2 diabetes; (3) progression to ESRD before renal biopsy; and (4) lack of trajectory of eGFR decline because serum creatinine levels were measured less than thrice annually at our hospital or performed at other hospitals (Supplementary Fig. 1).

In the untargeted proteomic study, six healthy control (HC) participants, six patients with T2DM, and 10 patients with T2DM and biopsy-proven DN were enrolled. In the targeted proteomic study, 29 HC participants, 22 patients with T2DM, and 54 patients with T2DM and biopsy-proven DN were enrolled. Trajectories of eGFR decline were obtained in the DN cohort during median follow-up duration of 47 months. All patients and HC participants provided written informed consent, and this study was approved by the institutional review board of the West China Hospital of Sichuan University. The study also complied with the 1964 Helsinki declaration and its later amendments or comparable ethical standards.

### Clinical and laboratory information

Clinical data including age, sex, and use of RAAS inhibitors, glucose-lowering agents, and statins were abstracted from electronic medical records from the time of renal biopsy to one of two endpoints: ESRD, or until April 30, 2020. Laboratory data at the time of renal biopsy were also obtained from medical records, including hemoglobin, serum creatinine, 24-h proteinuria, and urine albumin-to-creatinine ratio. eGFR was evaluated using the CKD Epidemiology Collaboration formula [21, 22]. Serum creatinine concentration was measured using the Cobas c702 chemistry autoanalyzer by enzymatic creatinine method (Roche Diagnostics, Rotkreuz, Switzerland). Subjects attended follow-up appointments two–four times annually, depending on their clinical condition. The primary endpoint of the study was progression to ESRD, indicated by eGFR < 15 mL/min/1.73 m^2^, or use of renal replacement therapy [22].

### Pathological features in DN

Biopsied renal tissue samples were routinely prepared for light microscopy, immunofluorescence, and electron microscopy using standard protocols at West China Hospital. Original immunofluorescence, microscopic, and electron microscopic images were used to confirm a diagnosis of DN. RPS classification and histological scoring [18, 19] under light microscopy or electron microscopy were evaluated by two nephropathologists, who were blinded to clinical data and renal outcomes.

### Urine sample collection and processing for proteomic analysis

Random urine samples were collected at the time of renal biopsy. Urine samples were centrifuged at 3500 rpm for 30 min to remove debris. The supernatant was collected in 5 mL tubes and then stored at −80 °C. Before proteomic analysis, 300 µL of supernatant from each urine sample was thawed. Following centrifugation at 13,000 rpm for 10 min, the supernatant was dried using a speed vac with a cold trap (CentriVap Cold Traps, Labconco, Kansas City, MO, USA). The protein concentration of each urine was measured using a Bradford protein assay (cat no. 500-0006, Bio-Rad, USA).

Urine containing 50 µg of protein was placed on 10 kDa polyethersulfone filters (PALL Life Science, USA) with 8 M urea (Sigma-Aldrich Corp., St. Louis, MO, USA). Protein was reduced with 20 mM dithiothreitol (Sigma-Aldrich Corp., St. Louis, MO, USA) and alkylated with 50 mM iodoacetamide (Sigma-Aldrich Corp., St. Louis, MO, USA). The solution was removed by centrifugation at 13,000*g* for 15 min and 200 µL of 50 mM ammonium bicarbonate (NH_4_CO_3_) (Sigma-Aldrich Corp., St. Louis, MO, USA) was added, and then digested with sequencing grade trypsin (Promega, Madison, WI, USA) at a ratio of 25:1 (*w/w*) for 16 h at 37 °C. The post-digestion peptide concentration was determined using a pierce quantitative colorimetric peptide assay kit (Thermo Fisher Scientific, Waltham, MA, USA).

Besides, an acetone precipitation method was also performed to deal with the same urine samples. The pre-cooled acetone (Sigma-Aldrich Corp., St. Louis, MO, USA) was added to the urine supernatant at a ratio of 3:1 (*V/V*) at −20 °C for 2 h. Then, the proteins were precipitated and were collected after centrifugation at 1000 rpm for 10 min, at 4 °C. The proteins were freeze dried and suspended in 100 µL of 50 mM NH_4_CO_3_. Protein was reduced with 20 mM dithiothreitol and alkylated with 50 mM iodoacetamide. Then, the sequencing grade trypsin was added to the mixture at a ratio of 25:1 (*w/w*) for incubating 16 h at 37 °C. After desalting using a pipette tip packed with a C18 membrane, the peptide concentration was determined using a pierce quantitative colorimetric peptide assay kit. The peptides were freeze dried for further analysis.

### Untargeted proteomics by data-dependent acquisition

The untargeted proteomics by data-dependent acquisition (DDA) analysis was performed according to a previous study [23]. Briefly, DDA was performed on an Orbitrap Fusion Lumos mass spectrometer (Thermo Fisher Scientific, Waltham, MA, USA) connected to an EASY-nLC 1200 system (Thermo Fisher Scientific, Waltham, MA, USA). The peptide separation was performed on an integrated spray-tip analytical column (75 μm i.d. × 25 cm) packed with 1.9 μm ReproSil-Pur 120 Å C18 resins (Dr. Maisch HPLC GmbH, Ammerbuch, Germany). A binary buffer system of 0.1% (*v/v*) formic acid (Sigma-Aldrich Corp., St. Louis, MO, USA) in water (buffer A) and 0.1% (*v/v*) formic acid and 80% acetonitrile (ACN) (Merck, KgaA, Darmstadt, Germany) in water (buffer B) was used for separation at a flow rate of 300 nL/min. The injection volume is 4 μL. A 78-min gradient was performed as follows: from 6 to 12% B in 8 min, from 12 to 28% B for 50 min, from 28 to 38% B for 12 min, from 38 to 95% B in 1 min, and held at 90% B for 7 min. The DDA method consisted of a full MS scan over m/z range of 350–1550 at a resolution of 120,000 in the Orbitrap mass analyzer followed by data-dependent MS/MS scans with a Top Speed method (3 s). MS/MS was carried out in the Orbitrap mass analyzer with a resolution of 15,000 using an isolation window of 2 *m/z* and high-energy collision-induced dissociation (HCD) fragmentation with normalized collision energy (NCE) of 35%. The dynamic exclusion time was set to 15 s. The Sequest HT node integrated within the Proteome Discoverer (PD) software (Version 2.1; Thermo Fisher Scientific, Waltham, MA, USA) was applied to search the raw data against the human Uniprot fasta database (70,947 entries, downloaded on Mar 10, 2017) appended with the Biognosys indexed retention time (iRT) peptides sequence. A maximum missed cleavages of two were allowed. Carbamidomethylation of cysteine (+ 57.02 Da) was chosen as static modification, while oxidation of methionine (+ 15.99 Da), deamidation of asparagine (+ 0.98 Da), and glutamine (+ 0.98 Da) were set as dynamic modifications. The first search mass tolerance was 20 ppm and the main search peptide tolerance was 4.5 ppm [24]. The false discovery rate (FDR) of peptide spectrum matches (PSMs) and proteins were set to < 1%. MaxQuant (Version 1.5.3.8; Max Planck Gesellschaft, Munich, Germany) was applied for the label-free quantification (LFQ) analysis of proteins with default settings. The same protein sequence database and the same modification parameters were used as the PD search. The FDR was controlled as 1% for both peptide spectrum matches and proteins.

### Targeted proteomics by PRM-MS

Proteins of interest were quantified using PRM-MS on an Orbitrap Fusion Lumos Tribrid mass spectrometer (Waltham, MA, USA) connected to an EASY-nLC 1200 system (Waltham, MA, USA). Peptides belong to 19 proteins were selected from untargeted proteomics. Best peptides by chromatographic data were selected for PRM-MS (Supplementary Table 1). Selected peptides were monitored by targeting precursor ions in the quadrupole analyzer (selection window 2.0 Da) and full-scan MS/MS after HCD fragmentation (NCE = 35%) in the Orbitrap analyzer with high resolution (15,000). Scheduled acquisition with 10-min acquisition windows was set up for each peptide precursor using Skyline (https://skyline.ms/project/home/software/skyline/begin.view), allowing a maximum of 30 concurrent PRM experiments. Acquired data were processed in Skyline software (version 3.7.0), and automated integration was manually checked. The identity of the chromatographic peaks was ascertained by matching the PRM-MS/MS spectra to those from the untargeted dataset (dot product 0.0.9 and mass precision, 5 ppm). Peptide and protein abundance data in the targeted proteomics were log10-transformed.

### Statistical analysis

Continuous variables are presented as mean and standard deviation (SD) if normally distributed, or as median and interquartile range (IQR) if non-normally distributed. Categorical variables are presented as counts and percentages. Differences in continuous variables between patients with progressive eGFR decline and slow eGFR decline were analyzed using the Student’s *t*-test or Wilcoxon test. Differences in continuous variables between HC participants, T2DM patients, and patients with T2DM and biopsy-proven DN were analyzed using one-way ANOVA, while categorical variables were analyzed using the chi-square test or Fisher’s exact test.

Correlations between the abundance of each urinary protein and baseline eGFR level, proteinuria, and hemoglobin A1c (HbA1c) were assessed using Spearman’s correlation analysis. Correlations between abundances of urinary proteins and annual eGFR decline treated as a continuous variable were initially assessed using Spearman’s correlation analysis. Univariate and two multivariable linear regression analyses were then used to assess the association between the abundance of urinary complement proteins and faster eGFR decline treated as a binary variable. The two multivariable regression analyses were adjusted for age, sex, baseline eGFR, and proteinuria. In the first multivariable regression analysis, each urinary complement protein was included individually in the model. Next, all significant proteins that were associated with faster eGFR decline in the first multivariable regression model were included in the second multivariable regression model. Parameters with *p* < 0.05 in the second adjusted multivariable regression model were considered to have a significant association with faster eGFR decline. Correlations between the abundance of each urinary protein and the pathological covariates were evaluated using linear regression analysis.

Survival curves were generated using Kaplan–Meier methods with a log-rank test. Univariate and multivariable Cox proportional hazards models were used to estimate the hazard ratios (HRs) of urinary complement proteins for renal outcome. The proportional hazard assumption in Cox models was tested to determine whether the dataset satisfied the basic assumptions of Cox analyses. Subsequently, Cox proportional hazards models were used to calculate HRs and 95% confidence intervals (CIs) for renal outcome. We applied three multivariable Cox proportional hazards models, all of which included clinical parameters (age, sex, baseline eGFR, proteinuria, and serum albumin concentration). The urinary abundance of each complement protein was included individually in the first two Cox proportional hazards models. Age and sex were selected based on biological plausibility. The clinical covariates were selected as potential confounders because of their significance in univariate analysis or bacause they were associated with ESRD in a previous study [25]. The second multivariable model incorporated the above parameters in addition to pathological parameters. Parameters with *p* < 0.05 in the second adjusted model were considered significant predictors of prognosis. Parameters with *p* < 0.05 in the second adjusted model were considered significant predictors of prognosis. Finally, a third multivariable Cox proportional hazards model, which included all proteins that individually remained significantly associated with ESRD in the second adjusted model, was constructed to assess which proteins represent prognostic risk factors for progression to ESRD. Receiver operating characteristic (ROC) curve analysis using clinical/pathological variables, prognostic urinary complement proteins, or these parameters in combination was applied to confirm and determine the best predictors of ESRD. The area under the curve (AUC) was calculated for each model [26].

All statistical analyses were performed using Stata version 14.0 (Stata Corp LLC, College Station, TX, USA). Statistical significance was accepted at *p* < 0.05. Other analyses were performed in R or with a customized in-house platform (https://www.omicsolution.org/wkomics/main/).

## Results

### Untargeted proteomics

**Fig. 1:**
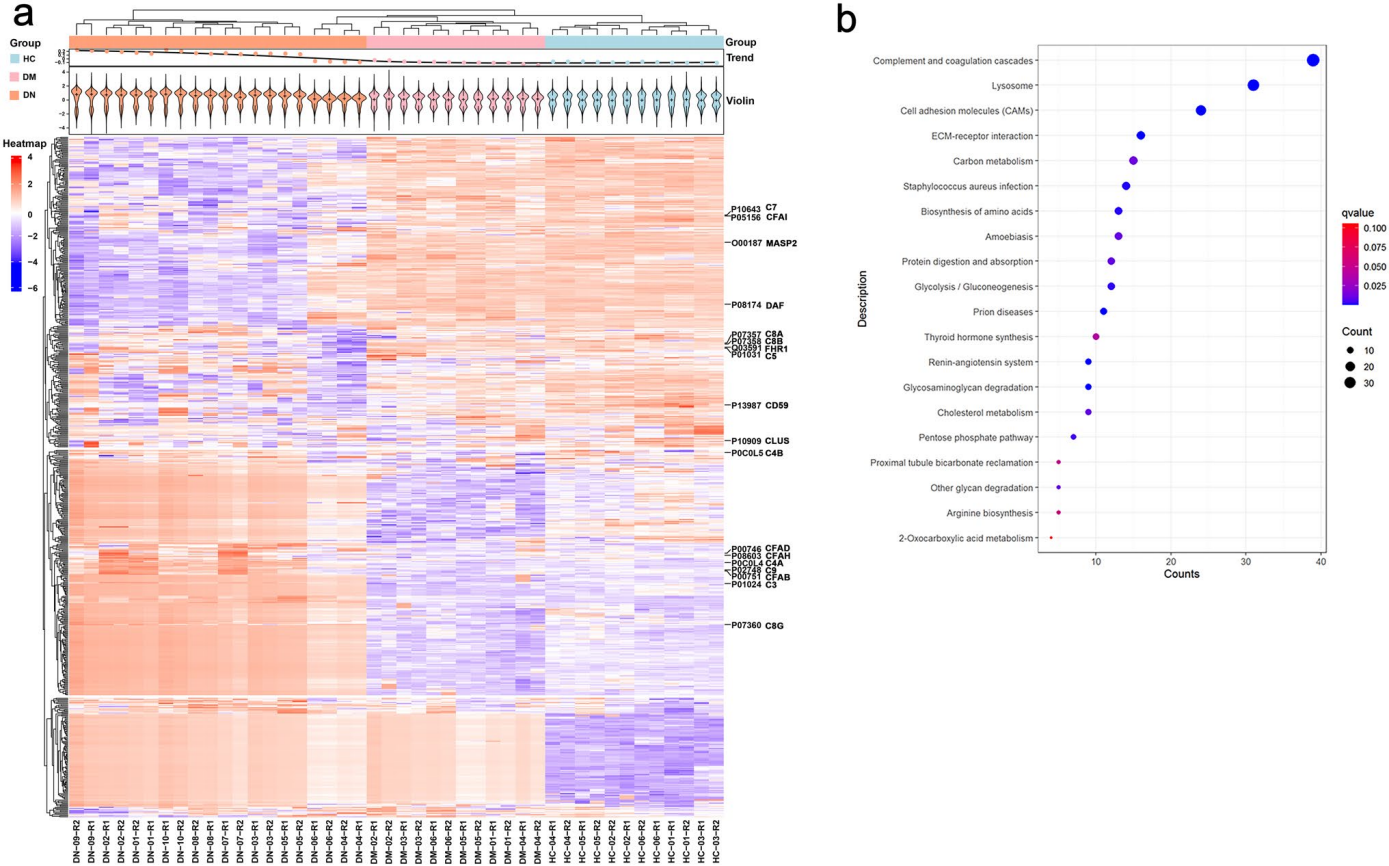
Urinary untargeted proteomic result by filter-aided proteome preparation method. **a** Hierarchical cluster analysis of the up-regulated and down-regulated urinary proteins in healthy control participants, patients with type 2 diabetes, patients with type 2 diabetes and DN. The *red bar* in the figure indicated the complement and complement regulatory proteins. **b** Functional analysis by KEGG showed that urinary proteins that differed in expression are linked to the complement and coagulation cascades. *KEGG* Kyoto Encyclopedia of Genes and Genomes

Demographics of participants in the untargeted proteomic study are shown in Supplementary Table 2. Compared with HC participants, patients with T2DM or DN had higher body mass index (BMI) but lower eGFR. In the untargeted proteomics study, of the 600 proteins identified by the aforementioned filter-aided proteome preparation method, 242 proteins were up-regulated and 272 were down-regulated in patients with T2DM and DN compared with those with T2DM. Hierarchical cluster analysis shows differences in expression of urinary proteins among the three groups (Fig. 1a). Functional analysis using the Kyoto Encyclopedia of Genes and Genomes (KEGG) showed that the urinary proteins that differed in expression were mostly linked to the complement and coagulation cascades (Fig. 1b). This observation was also verified by the acetone precipitation method (Supplementary Fig. 2).

### Targeted proteomics

#### Participants features

Among patients with T2DM and DN, clinical and pathological features were similar between the 54 patients who were enrolled and the 89 patients who were not enrolled in the targeted proteomic study (Supplementary Table 3). The demographic and clinical features of participants, including HC participants, T2DM patients, and patients with T2DM and DN in the subsequent targeted proteomics study are shown in Supplementary Table 4. Average age among the three groups was comparable. In the T2DM and DN group, the age (mean ± SD) was 52 ± 9 years, and 32 were men. Median duration of diabetes was 108 months (IQR, 48–168). Median baseline eGFR was 71.8 mL/min/1.73 m^2^, which was significantly lower than in HC participants or patients with T2DM (*p* < 0.001). All patients with T2DM and DN underwent at least three annual measurements of serum creatinine or eGFR. During median follow-up duration of 47 months, annual eGFR decline in the DN cohort was −7.99 mL/min/1.73 m^2^ (IQR −15.75 to −1.09). According to the median annual rate in eGFR decline, 26 patients demonstrated slow decline in eGFR, whereas 28 patients demonstrated progressive decline in eGFR. Median annual eGFR decline was −15.70 mL/min/1.73 m^2^ (IQR −23.28 to −11.06) in progressive eGFR decliners and −1.07 mL/min/1.73 m^2^ (IQR −3.70 to 1.98) in slow eGFR decliners (*p* < 0.001). Age, sex distribution, BMI, blood pressure, and baseline eGFR were comparable between slow and progressive eGFR decliners. Additionally, there were no significant differences in medication use between progressive and slow eGFR decliners. However, the diabetes duration was shorter and baseline proteinuria was higher in progressive eGFR decliners compared with slow eGFR decliners. Furthermore, compared with slow decliners, progressive decliners had higher percentages of RPS classes IIb and III, and lower percentages of class I. Progressive eGFR decliners had higher interstitial fibrosis and tubular atrophy (IFTA) scores than slow eGFR decliners (Table 1).

**Table 1:** The clinical characteristics of patients stratified by eGFR decline rate

Characteristics	Slow eGFR decliners (*n* = 26)	Progressive eGFR decliners (*n* = 28)	*p* value
Age, mean (SD), y	53 (9)	52 (9)	0.89
Sex, Male, *n* (%)	17 (65.4)	15 (53.6)	0.44
Smoking, Never/Ex/Current, (*n*)	14/3/9	20/2/6	0.32
BMI, mean (SD), kg/m^2^	25.8 (3.2)	24.4 (3.1)	0.43
SBP, mean (SD), mmHg	138 (21)	141 (27)	0.34
DBP, mean (SD), mmHg	81 (13)	84 (12)	0.71
Duration of diabetes, median (IQR), months	132 (96–180)	66 (36–133)	0.04
HbA1c, median (IQR), %	7.6 (6.8–7.9)	7.5 (6.6–9.2)	0.72
FPG, median (IQR), mg/dL	151.6 (112.3–194.9)	135.8 (99.4–161.6)	0.69
Hemoglobin, mean (SD), g/L	133.7 (24.7)	125.5 (24.0)	0.05
Serum albumin, mean (SD), g/L	41.8 (4.7)	35.6 (7.1)	< 0.001
eGFR, median (IQR), mL/min/1.73 m^2^	67.1 (46.2–92.3)	78.2 (50.2–94.8)	0.37
24-h proteinuria, median (IQR), g/d	1.44 (0.53–2.74)	3.48 (1.50–7.6)	< 0.001
uACR, median (IQR), (mg/g)	739 (164–1488)	1727 (936–3692)	0.04
RAAS inhibitors, No. (%)	23 (88.5)	25 (89.3)	0.90
OHA therapy, No. (%)	15 (57.7)	15 (53.6)	0.76
Insulin therapy, No. (%)	19 (73.1)	18 (64.3)	0.48
Statins, No. (%)	17 (65.4)	19 (67.9)	0.84
RPS classification^a^, *n* (%)			0.04
I	5 (19.2)	2 (7.1)	
IIa	6 (23.1)	6 (21.4)	
IIb	4 (15.4)	5 (17.9)	
III	9 (34.6)	12 (42.9)	
IV	2 (7.7)	3 (10.7)	
IFTA^a^, *n* (%)			0.04
Score 0	3 (11.5)	2 (7.1)	
Score 1	15 (57.7)	14 (50.0)	
Score 2	7 (26.9)	10 (35.7)	
Score 3	1 (3.8)	2 (7.1)	
Interstitial inflammation^a^, *n* (%)			0.22
Score 0	0 (0)	0 (0)	
Score 1	20 (76.9)	25 (89.3)	
Score 2	6 (23.1)	3 (10.7)	
Arteriosclerosis^a^, *n* (%)			0.68
Score 0	3 (11.5)	3 (10.7)	
Score 1	13 (50.0)	11 (39.3)	
Score 2	10 (38.5)	14 (50.0)	
Arteriolar hyalinosis^a^, *n* (%)			0.60
Score 0	1 (3.8)	3 (10.7)	
Score 1	10 (38.5)	9 (32.1)	
Score 2	15 (57.7)	16 (57.1)	

*BMI* body mass index; *SBP* systolic blood pressure; *DBP* diastolic blood pressure; *FPG* fasting plasma glucose; *eGFR* estimated glomerular filtration rate; *HbA1c* hemoglobin A1c; *uACR* urine albumin-to-creatinine ratio; *RAAS* renin–angiotensin–aldosterone system; *OHA* oral hypoglycemic agent; *RPS* Renal Pathology Society; *IFTA* interstitial fibrosis and tubular atrophy

^a^Defined by RPS DN classification. Data are presented as means (SDs) for continuous variables with a normal distribution, as medians (25th–75th percentiles) for continuous variables without a normal distribution, and as percentages for categorical variables

#### 3.2.2 Distributions of urinary complement proteins among control participants, patients with type 2 diabetes, and diabetic patients with associated DN

**Fig. 2:**
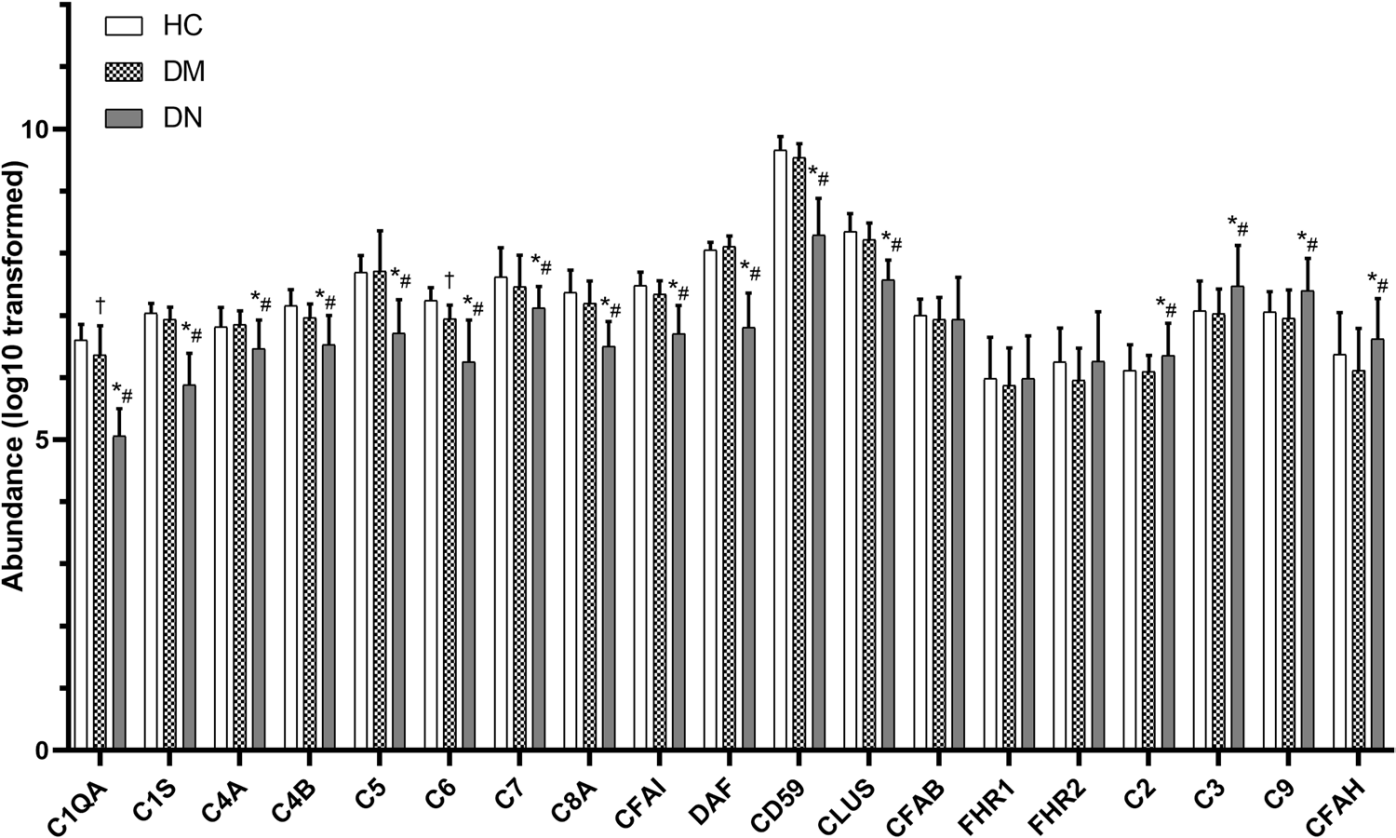
Distributions of urinary complement proteins among control participants, patients with type 2 diabetes, and diabetic patients with associated DN. *DN* diabetic nephropathy

Distributions of urinary complement proteins per unit of total urinary protein (i.e., the fraction of total urinary protein composed of a given complement protein) among HC participants, patients with T2DM, and patients with T2DM and DN are shown in Fig. 2. Compared with HC participants and patients with T2DM, patients with T2DM and DN had higher urinary abundance of complement C2, C3, and C9 but lower urinary abundance of complement C1QA, C1S, C4A, C4B, C5, C6, C7, and C8A. Among complement regulatory proteins, urinary abundance of complement factor H (CFAH) was significantly higher, whereas complement factor I (CFAI), decay-accelerating factor (DAF, also known as CD55), CD59, and clusterin (CLUS) were significantly lower in patients with T2DM and DN compared with HC participants or patients with T2DM. The urinary abundance of complement factor B, factor H-related protein 1 (FHR1), and FHR2 was similar among the three groups.

When stratified by annual rate of eGFR decline, patients with T2DM and DN patients with progressive eGFR decline had significantly higher levels of urinary complement C2, C3, and C9 than those with slow eGFR decline. Urinary complement regulatory proteins such as CFAH were significantly higher, whereas urinary DAF and CD59 were significantly lower in progressive eGFR decliners compared with slow eGFR decliners. Urinary abundance of C1QA, C1S, C4A, C4B, C5, C6, C7, and C8A, and complement regulatory protein CFAI, CFAB, FHR1, and FHR2 were not significantly different between patients with progressive or slow eGFR decline.

#### Correlation between urinary complement proteins by targeted proteomics and indices of diabetic kidney injury

**Fig. 3:**
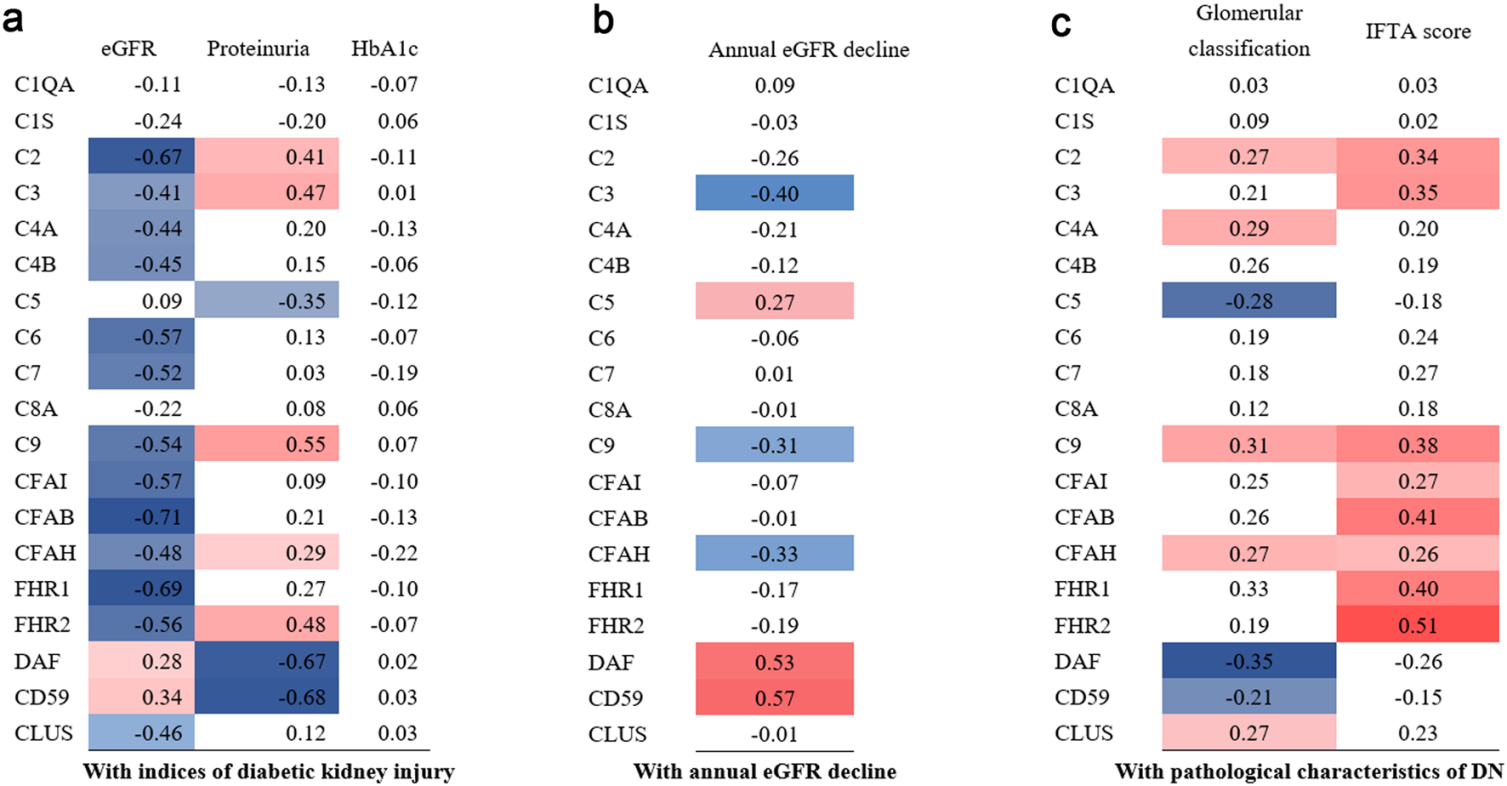
Matrix of correlation coefficients. **a** Matrix of correlation coefficients between urinary complement proteins and baseline parameters of diabetic kidney injury by Spearman’s correlation analysis. **b** Matrix of correlation coefficients between urinary complement proteins and annual eGFR decline during the follow-up period. **c** Matrix of correlation coefficients between urinary complement proteins and pathological parameters of DN by linear regression analysis. Cell color indicates the strengths and directions of the correlation from *blue* (negative correlation) to *white* (no correlation) to *red* (positive correlation). *DN* diabetic nephropathy

A matrix of the correlation coefficients between complement proteins and indices of diabetic kidney injury is showed in Fig. 3. 79% (15/19) of the urinary complement proteins were associated with baseline eGFR (Fig. 3a). The abundances of urinary complement C2, C3, C4A, C4B, C6, C7, C9, CFAI, CFAB, CFAH, FHR1, and FHR2 were significantly negatively correlated with baseline eGFR (Spearman correlation coefficient [*r*] =  −0.41 to −0.71). The abundances of urinary complement C2, C3, C9, CFAH, and FHR2 were significantly positively correlated with baseline proteinuria (*r* = 0.29–0.48). Urinary abundances of complement regulatory proteins DAF and CD59 showed a positive correlation with baseline eGFR (*r* = 0.28 and 0.34, respectively), and a negative correlation with baseline proteinuria (*r* =  −0.67 and −0.68, respectively). However, no urinary complement proteins were significantly associated with baseline HbA1c (Fig. 3a).

We next analyzed the association between the urinary abundance of complement proteins and annual eGFR decline in patients with T2DM and DN. Spearman’s correlation analysis showed that urinary abundances of C3, C9, and CFAH were negatively correlated with annual eGFR decline (*r* =  −0.31 to −0.40), while the urinary abundance of C5, DAF, and CD59 was positively correlated with annual eGFR decline treated as a continuous variable (*r* = 0.27–0.57) (Fig. 3b). Multiple regression analysis to assess the association between the abundance of urinary complement proteins and faster eGFR decline treated as a binary variable also confirmed this result (Supplementary Table 5). A further multivariable regression model, which included all proteins that were significantly associated with faster eGFR decline, showed that high abundance of urinary CFAH was a significant independent predictor of faster eGFR decline (standardized coefficient (*β*) 0.20, 95% CI 0.03–0.34, *p* = 0.03). Low abundances of urinary DAF and CD59 were also significant independent predictors of faster eGFR decline (*β* −0.47, 95% CI −0.79 to −0.06, *p* = 0.02; *β −*0.40, 95% CI −0.55 to −0.14, *p* < 0.01, respectively).

#### Correlation between uriary complement proteins by targeted proteomics and baseline renal pathological findings

Linear regression analysis showed that RPS glomerular classifications were significantly associated with high abundance of urinary complement C2, C4A, C9, CFAH, and CLUS (*R*^2^ = 0.23–0.99, standard *β* = 0.28–0.31), and low abundance of urinary DAF and CD59 (*R*^2^ = 0.17, standard *β* =  −0.35; *R*^2^ = 0.11 standard *β* =  −0.21, respectively) (Fig. 3c). IFTA scores were significantly associated with high abundance of urinary complement C2, C3, C9, CFAI, CFAB, CFAH, FHR1, and FHR2 (*R*^2^ = 0.08–0.12, standard *β* = 0.26–0.51). The abundance of urinary DAF and CD59 were not significantly associated with IFTA scores. The severity of interstitial inflammation, arteriolar hyalinosis, or arteriosclerosis was not significantly correlated with any urinary complement protein (data not shown).

#### Complement proteins and risk of ESRD

**Fig. 4:**
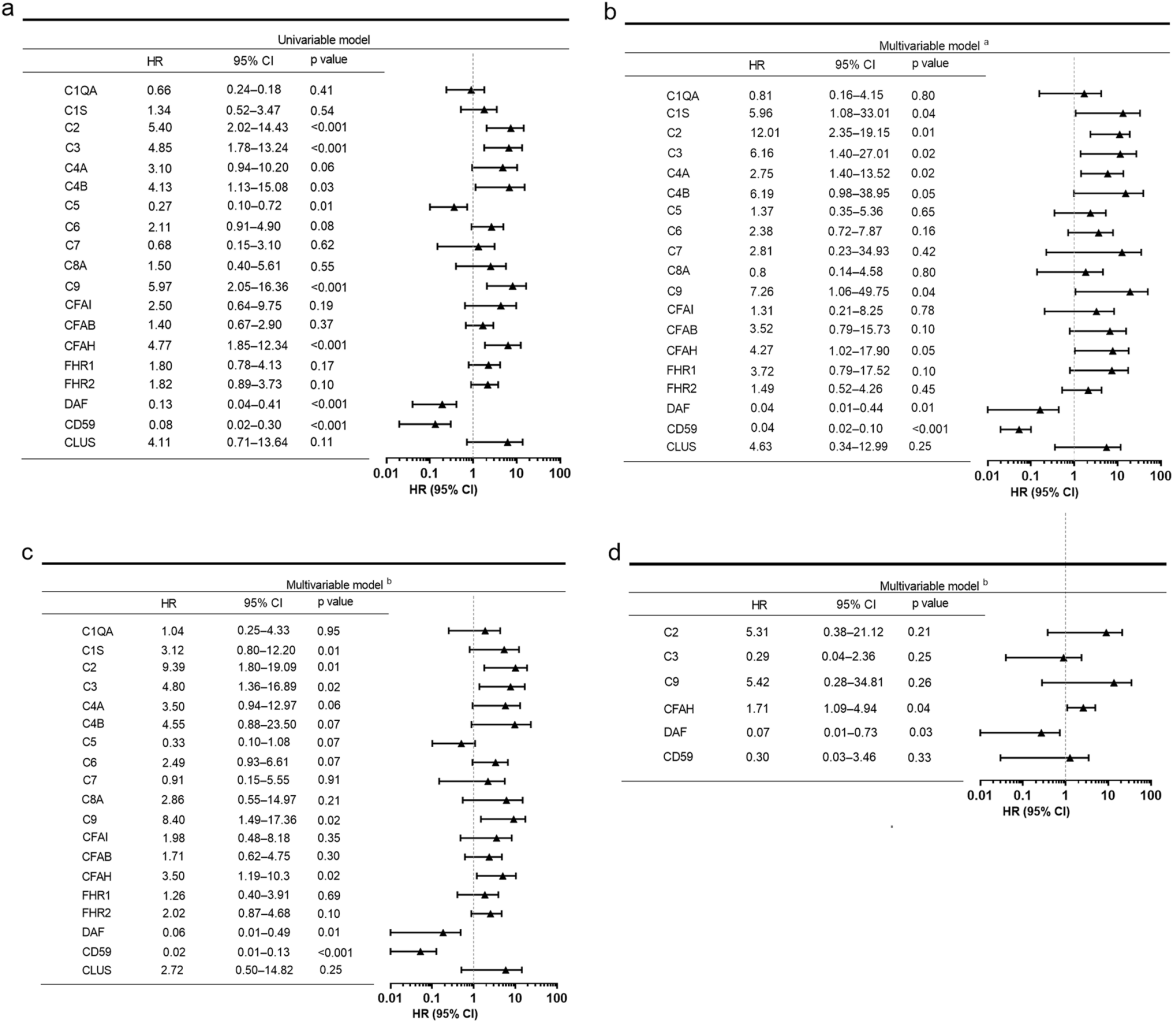
Association of urinary complement proteins with renal outcome in patients with type 2 diabetes and DN. Univariable (**a**) and two multivariable (**b**, **c**) Cox proportional hazards models by individual complement proteins at the renal endpoint. (**d**) The third multivariable Cox proportional hazards model included all proteins that were individually significantly associated with ESRD in the second multivariable model. ^a^Adjusted for age, sex, baseline log2 (baseline eGFR) and log2 (baseline urinary protein concentration); ^b^Adjusted for age, sex, baseline log2 (baseline eGFR) and log2 (baseline urinary protein concentration), Renal Pathology Society glomerular classification, interstitial fibrosis and tubular atrophy. *CFAI* complement factor I; *CFAB* complement factor B; *CFAH* complement factor H; *FHR1* factor H-related protein 1; *FHR2* factor H-related protein 2; *DAF* decay-accelerating factor; *CLUS* clusterin

Of patients with T2DM and DN at risk, 35.2% (19/54) progressed to ESRD. The urinary abundance of each quantified complement protein was included individually in Cox proportional hazards models for predicting progression to ESRD (Fig. 4). Univariable Cox proportional hazards analysis showed that urinary complements C2, C3, C4B, C5, C9, CFAH, DAF, and CD59 were associated with future ESRD (Fig. 4a). After adjusting for age, sex, baseline eGFR, and proteinuria, higher abundances of urinary complement C1s, C2, C3, C4A, C4B, C9, and CFAH, but lower abundances of urinary DAF and CD59 were significantly associated with higher risk of progression to ESRD (Fig. 4b). However, after further adding renal pathological covariates in the second multivariable Cox proportional hazards model, only higher abundances of urinary complement C2, C3, C9, and CFAH were associated with higher risk (adjusted HR, range from 3.50 to 9.39) of progression to ESRD, while lower abundance of urinary DAF (adjusted HR, 0.06) or CD59 (adjusted HR, 0.02) was significantly associated with higher risk of progression to ESRD (Fig. 4c). Adding diabetes duration or baseline HbA1c did not alter these findings significantly (data not shown). A third multivariable Cox proportional hazards model, which included all proteins that were individually significantly associated with ESRD in the second multivariable model, was then performed. Higher abundance of urinary CFAH (adjusted HR, 1.70) but lower abundance of urinary DAF (adjusted HR, 0.07), was significantly associated with higher risk of progression to ESRD (Fig. 4d) in patients with T2DM and DN.

**Fig. 5:**
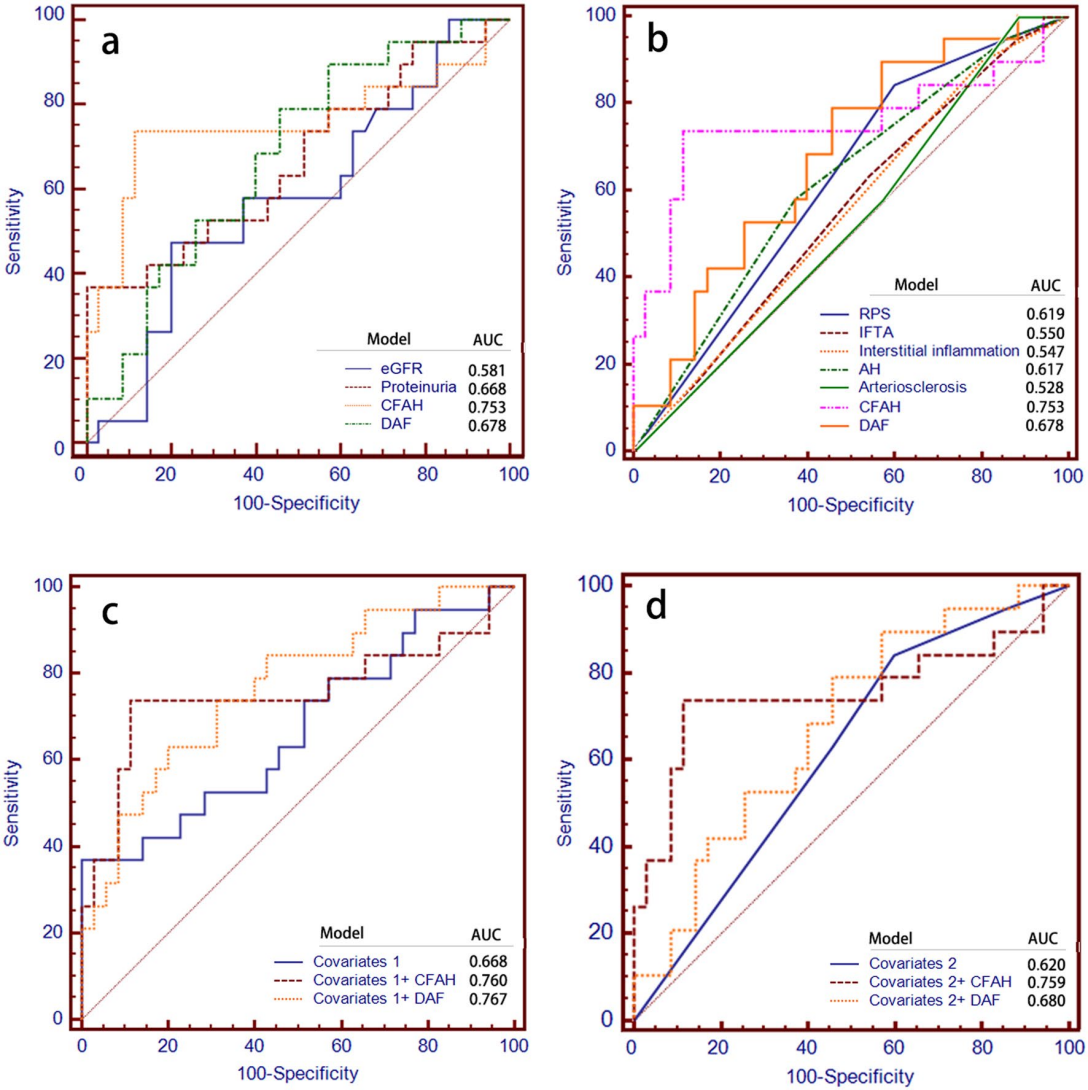
Area under the receiver operating characteristic curve for prediction of ESRD. Urinary abundance of CFAH and DAF had a larger AUC than clinical (**a**) or pathological parameters (**b**). The model contains covariates and CFAH or DAF had lager AUC than that of the model that only contains clinical parameters (**c**) or only pathological parameters (**d**). Covariates 1 were baseline eGFR and proteinuria. Covariates 2 were RPS glomerular classification, IFTA, interstitial inflammation, arteriosclerosis, and AH. *ESRD* end-stage renal disease; *AUC* area under the curve; *CFAH* complement factor H; *DAF* decay-accelerating factor; *eGFR* estimated glomerular filtration rate; *RPS* Renal Pathology Society; *IFTA* interstitial fibrosis and tubular atrophy; *AH* artery hyalinosis

The ROC AUC for prediction of ESRD according to clinical and pathological parameters, and urinary complement proteins CFAH or DAF are shown in Fig. 5. Urinary abundance of CFAH and DAF had a larger AUC than that for eGFR and proteinuria (Fig. 5a). Moreover, the ROC AUC for urinary abundance of CFAH and DAF was larger than that for any pathological parameter (Fig. 5b). The model that included CFAH or DAF had a larger AUC than the model with only clinical parameters (eGFR and proteinuria) or only pathological parameters (RPS classification, IFTA, interstitial inflammation, arteriosclerosis, and artery hyalinosis) (Fig. 5c, d).

## Discussion

Targeted and untargeted proteomic studies revealed abnormal abundances of urinary complement proteins in patients with T2DM and DN compared with those with T2DM and HC participants. In patients with T2DM and DN, urinary complement C2, C9, CFAH, DAF, CD59, and CLUS correlated strongly with RPS glomerular classification, while C2, C3, C9, and CFAH correlated well with IFTA. Multivariable Cox proportional hazards analysis revealed that higher urinary abundance of complement CFAH, and lower urinary abundance of DAF were associated with greater risk of progression to ESRD when adjusting for age, sex, baseline renal function, and renal pathological covariates, including RPS glomerular classification and IFTA score. Urinary abundance of CFAH and DAF had a larger AUC than that of eGFR, proteinuria, or any pathological parameter. Moreover, the model that included CFAH or DAF had a larger AUC than the model with only clinical or pathological parameters, indicating that urinary abundance of CFAH and DAF provided added value for predicting progression to ESRD in patients with T2DM and biopsy-proven DN.

The complement system serves as part of the innate immune system required for host defense, and is activated via the classical, alternative, or mannose-binding lectin pathways [27, 28]. Inappropriate activation of the complement pathway leads to kidney damage [28]. Recent evidence indicates that complement activation is associated with development of glomerular and tubulointerstitial injury [29–31]. Complement system-mediated progression of renal disease may occur via both immune and non-immune pathways [27]. Changes of urinary complement proteins may reflect complement overproduction, secretion, and/or deposition in the diabetic injured kidney [32].

A key finding of this study was that higher urinary abundance of CFAH was associated with higher risk of onset of ESRD in patients with T2DM and biopsy-proven DN. CFAH, an abundant serum glycoprotein, is one of the most important circulating regulators of the alternative pathway. It is expressed constitutively in liver and locally expressed by endothelial cells, epithelial cells, and podocytes [33]. CFAH serves as an essential cofactor for CFAI-mediated C3b cleavage. It also accelerates decay of the alternative pathway C3 convertase [34]. Clinical studies showed an association between CFAH polymorphisms and adverse clinical outcomes in diabetic patients [35, 36]. Experimental studies addressed the fact that in the absence of CFAH, spontaneous activation of the alternative pathway of complement occurs in plasma, which leads to consumption of complement C3, secondary plasma C3 deficiency, and massive accumulation of C3 along the glomerular basement membrane [37]. In cultured immortalized human podocytes and primary human podocytes from a known Arg1182Ser (G3546T) CFAH mutant patient, CFAH was confirmed to be expressed by podocytes. Moreover, expression of CFAH was up-regulated in injured podocytes [33]. As a molecule of 150 kDa, CFAH is prevented from filtration by a healthy glomerular filtration barrier. Our findings did not identify the source of urinary CFAH. It may have been derived from injured podocytes or epithelial tubular cells, or filtered through an impaired glomerular filtration barrier [38]. If high urinary CFAH level was a consequence of increased renal CFAH production, its association with greater risk of progression to ESRD indicates insufficient CFAH-mediated protection from proteinuria-induced complement activation, resulting in progressive eGFR decline and ultimately, ESRD. In contrast, the strong association between urinary CFAH excretion and proteinuria suggests high urinary CFAH because impaired glomerular permeability cannot be excluded.

DAF is a glycophosphatidylinositol-anchored protein expressed both on podocytes and epithelial tubular cells [39] that regulates the C3 and C5 convertases of the classical and alternative pathways [40]. DAF accelerates decay and inhibits the reformation of surface-bound C3 convertases, together with retraining amplification of the cascade [39]. In streptozotocin-induced diabetic glomerulosclerosis, podocyte-specific deficiency of DAF activated C3a/C3aR signaling, causing actin cytoskeleton rearrangement and podocyte injury [39]. Furthermore, DAF mRNA and protein expression were down-regulated in glomeruli in diabetes [39, 41]. A streptozotocin-induced diabetic animal model showed increased DAF cleavage on the podocyte membrane, resulting in low levels of DAF in renal tissue and urine [39]. Combined with previous studies, the association between lower abundance of urinary DAF and higher risk of ESRD in our DN cohort indicated an important protective effect on kidney disease progression exerted by DAF. In the present study, although CD59 was not an independent risk factor for predicting future ESRD, it predicted faster eGFR decline in patients with T2DM and DN. CD59 is a transmembrane protein that inhibits formation of the membrane attack complex (MAC), thereby protecting cells from complement-mediated injury. Vaisar et al*.* reported that lower urinary CD59 was an independent predictor of higher risk of future ESRD and all-cause mortality in a Mexican–American cohort with T2DM and CKD [32]. Transgenic mice expressing human DAF and CD59 had reduced C3 and C9 deposition in the kidney and inhibition of terminal complement pathway [42], suggesting that targeting DAF and CD59 may prevent renal injury.

In this study, higher levels of urinary complement C2 and C3 were significantly associated with higher risk of future ESRD by multivariable Cox proportional hazards analysis. This result suggests a degree of activity from the lectin and classical complement pathways may be associated with shorter renal survival in patients with T2DM and DN. Our previous study using single-nephron proteomic analysis and immunohistochemical staining suggested complement factors such as C3, C8, and C9 are overactivated in solidified glomerulosclerosis in patients with DN. Solidified glomerulosclerosis was shown to be an independent pathological finding that predicts future diabetic ESRD [43]. C9 binds C8 in the C5b-8 complex and forms the MAC that causes cellular and organ damage. Previous studies showed that urinary MAC excretion correlates well with eGFR decline in DN [44, 45], while glomerular MAC deposition correlates with the severity of DN and microvascular and interstitial lesions [46]. Therefore, a promising approach to delaying progression of DN may be to block the lectin and classical pathways by targeting C2 or C3, or by inhibiting MAC formation by targeting C9.

This study revealed that patients with progressive eGFR decline had more severe pathological features than those with slow eGFR decline. Early validation studies showed that higher-grade lesions, assessed using the RPS classification, were associated with greater risk of ESRD and requirement for dialysis in patients with T2DM [15, 18, 47]. A strong structural–functional relationship existed in both T1DM and T2DM. A Japanese nationwide multicenter study that analyzed kidney biopsy samples found that pathological features, including glomerular, interstitial, and vascular lesions, were more severe in high-risk categories and were highest in the red group (based on the CKD heatmap category according to baseline eGFR and albuminuria level) [48]. Although diabetes duration was shorter in rapid eGFR decliners than slow eGFR decliners, the association between duration of diabetes and eGFR decline was controversial. Previous studies showed that the differences in duration between progressors and nonprogressors were similar [26, 49, 50]. A cross-sectional study of 17 patients with T2DM and DN showed that histopathological changes were not significantly associated with duration of diabetes [51]. The absence of an association between renal structural alterations and diabetic duration may be explained by the high heterogeneity and insidious onset of T2DM. Numerous individuals have undiagnosed diabetes or impaired glucose tolerance for extended periods of time, which leads to inaccurate assessment of diabetes duration [52]. Many patients have delayed diagnosis and treatment of diabetes. Lack of disease awareness in these patients may lead to rapid progression of kidney injury. This observation reemphasizes the importance of early screening and intervention for delaying progression of diabetes.

An advantage of this study was the comprehensive quantification of urinary complement proteins using a targeted mass spectrometric method. Patients had pathologic diagnosis of diabetic glomerulosclerosis, and we excluded those with evidence of any other glomerular disease. The correlation between urinary complement proteins and renal outcome was carefully adjusted for demographic parameters, clinical covariates, and pathological confounders. A limitation of the study was the limited number of patients with biopsy-proven DN. Additionally, quantification of complement protein expression in serum and kidney tissue was not performed. Given our results, we propose alteration of urinary complement proteins may be associated with more severe inflammatory status and may lead to poor renal outcome in patients with T2DM and DN, This implies therapeutically targeting the complement system may help improve the renal outcome of DN in clinical practice. Nonetheless, the specific mechanism underlying overactivation of the complement system need more experimental and clinical evidence to verify.

In conclusion, compared with patients with T2DM and HC participants, the complement system was overactivated in patients with T2DM and DN, and was reflected as abnormal urinary excretion of complement proteins. Higher abundance of urinary CFAH was strongly associated with higher risk of progression to ESRD, while lower abundance of DAF was associated with greater risk of future ESRD. These findings indicated that activation of different arms of the complement pathway may be associated with higher risk of progression to ESRD. Consequently, therapeutically targeting the complement pathway may alleviate progression of DN.

## Supplementary Information

Below is the link to the electronic supplementary material.

Supplementary file1 (DOCX 698 KB)

## Data Availability

The original contributions presented in the study are included in the article/supplementary materials, further inquiries can be directed to the corresponding author/s.
