# Supplementary material for: Urinary complement proteins and risk of end-stage renal disease: quantitative urinary proteomics in patients with type 2 diabetes and biopsy-proven diabetic nephropathy
Source: J Endocrinol Invest. 2021 May 27;44(12):2709–23. doi: 10.1007/s40618-021-01596-3 (PMC8572220; doi:10.1007/s40618-021-01596-3)
Supplement: Supplementary file 1 — Supplementary file1 (DOCX 698 KB) [file 40618_2021_1596_MOESM1_ESM.docx]

**Supplementary Table S1. Peptides used to quantify the targeted proteins**

| Protein Name | Protein Accession | Peptide Sequence | Precursor m/z | Precursor Charge | Fragment Ions (Charge) |
| --- | --- | --- | --- | --- | --- |
| Complement C1QA | P02745 | GTKGSPGNIK | 479.7694 | 2+ | y2(1+), b7(1+), b8(1+) |
| Complement C1S | P09871 | SSNNPHSPIVEEFQVPYNK | 729.3553 | 3+ | y4(1+), y2(1+), b9(1+) |
|  |  | EDTPNSVWEPAK | 686.8226 | 2+ | y9(1+), y5(1+), y3(1+) |
|  |  | VEDPESTLFGSVIR | 774.8988 | 2+ | y11(1+), y9(1+), y6(1+) |
| Complement C2 | P06681 | SSGQWQTPGATR | 638.3075 | 2+ | y7(1+), y6(1+), y5(1+) |
|  |  | AVCKPVR | 415.2393 | 2+ | y5(1+), y4(1+), y3(1+) |
|  |  | AVISPGFDVFAK | 625.8426 | 2+ | y9(1+), y8(1+), y8(2+) |
| Complement C3 | P01024 | SNLDEDIIAEENIVSR | 908.9498 | 2+ | y9(1+), y8(1+), b2(1+) |
|  |  | ILLQGTPVAQMTEDAVDAER | 1079.046 | 2+ | y14(1+), y12(1+), y3(1+) |
|  |  | SGSDEVQVGQQR | 645.3077 | 2+ | y7(1+), y6(1+), y4(1+) |
| Complement C4A | P0C0L4 | ANSFLGEK | 433.2243 | 2+ | y6(1+), y4(1+), y3(1+) |
| Complement C4B | P0C0L5 | ASSFLGEK | 419.7189 | 2+ | y6(1+), y4(1+), y3(1+) |
|  |  | AEMADQAAAWLTR | 725.3432 | 2+ | y10(1+), y8(1+), y7(1+) |
| Complement C5 | P01031 | FSDASYQSINIPVTQNMVPSSR | 819.7287 | 3+ | y4(1+), y2(1+), y11(2+) |
|  |  | EGMLSIMSYR | 593.7834 | 2+ | y2(1+), y1(1+), b2(1+) |
| Complement C6 | P13671 | CPINCLLGDFGPWSDCDPCIEK | 885.0445 | 3+ | b3(1+), b3(2+), b4(2+) |
|  |  | IGESIELTCPK | 623.821 | 2+ | y10(1+), y8(1+), y6(1+) |
|  |  | GFVVAGPSR | 445.2482 | 2+ | y6(1+), y5(1+), b2(1+) |
| Complement C7 | P10643 | ECNNPPPSGGGR | 621.27 | 2+ | y8(1+), y7(1+), y6(1+) |
|  |  | ILPLTVCK | 472.2859 | 2+ | y6(1+), y4(1+), y2(1+) |
|  |  | EQTMSECEAGALR | 741.3216 | 2+ | y11(1+), y9(1+), y7(1+) |
| Complement C8A | P07357 | KPYNFLK | 455.2633 | 2+ | y1(1+), b1(1+), b2(1+) |
| Complement C9 | P02748 | VVEESELAR | 516.272 | 2+ | y7(1+), y6(1+), y5(1+) |
|  |  | FTPTETNK | 469.2349 | 2+ | y6(1+), y5(1+), y6(2+) |
|  |  | AIEDYINEFSVR | 728.3594 | 2+ | y10(1+), y7(1+), y6(1+) |
| Complement factor I | P05156 | HGNTDSEGIVEVK | 692.8388 | 2+ | y2(1+), b8(1+), b9(1+) |
|  |  | GLETSLAECTFTK | 728.853 | 2+ | y9(1+), y7(1+), y6(1+) |
|  |  | VFSLQWGEVK | 596.8217 | 2+ | y8(1+), y5(1+), y4(1+) |
| Complement factor B | P00751 | STGSWSTLK | 483.7482 | 2+ | y7(1+), y4(1+), b2(1+) |
|  |  | VSEADSSNADWVTK | 754.8468 | 2+ | y10(1+), y9(1+), y4(1+) |
|  |  | GDSGGPLIVHK | 540.2958 | 2+ | y6(1+), y3(1+), y2(1+) |
| Complement factor H | P08603 | TGDEITYQCR | 621.7746 | 2+ | y6(1+), y5(1+), b3(1+) |
|  |  | LSYTCEGGFR | 595.269 | 2+ | y9(1+), y7(1+), y6(1+) |
|  |  | CFEGFGIDGPAIAK | 741.3583 | 2+ | y11(1+), y9(1+), y6(1+) |
| Complement factor H-related protein 1 | Q03591 | TGESAEFVCK | 564.2555 | 2+ | y6(1+), y5(1+), y2(1+) |
| Complement factor H-related protein 2 | P36980 | ITCAEEGWSPTPK | 738.3454 | 2+ | y10(1+), y9(1+), y7(1+) |
|  |  | TGDIVEFVCK | 584.2894 | 2+ | y6(1+), y5(1+), b3(1+) |
|  |  | LVYPSCEEK | 562.7683 | 2+ | y7(1+), y6(1+), y6(2+) |
| Complement decay-accelerating factor | P08174 | TSFPEDTVITYK | 700.8508 | 2+ | y9(1+), y8(1+), y9(2+) |
|  |  | LNSASLK | 366.7162 | 2+ | y6(1+), y3(1+), y2(1+) |
|  |  | EIYCPAPPQIDNGIIQGER | 1085.534 | 2+ | y15(1+), y13(1+), y13(2+) |
| CD59 glycoprotein | P13987 | AGLQVYNK | 446.748 | 2+ | y4(1+), y3(1+), y2(1+) |
|  |  | FEHCNFNDVTTR | 513.893 | 3+ | y5(1+), y4(1+), y3(1+) |
|  |  | ENELTYYCCK | 690.2839 | 2+ | y6(1+), y4(1+), y3(1+) |
| Clusterin | P10909 | TLLSNLEEAK | 559.3086 | 2+ | y8(1+), y7(1+), y6(1+) |
|  |  | IDSLLENDR | 537.7749 | 2+ | y8(1+), y7(1+), y5(1+) |
|  |  | FMETVAEK | 477.7337 | 2+ | y7(1+), y6(1+), y3(1+) |

**Supplementary Table S2. Baseline characteristics of study participants**

| Characteristics | HC (n=6) | DM (n=6) | DN (n=10) | *p* value |
| --- | --- | --- | --- | --- |
| Age, mean (SD), y | 51 (8) | 54 (8) | 52 (9) | 0.23 |
| Sex, Male, n (%) | 3 (50.0) | 3 (50.0) | 6 (60.0) | 0.14 |
| Smoking, Never/Ex/Current, (n) | 1/3/2 | 0/3/3 | 4/2/4 | 0.36 |
| BMI, mean (SD), kg/m^2^ | 22.3 (2.8) | 24.1 (2.6) | 25.7 (2.9) | <0.001 |
| Duration of diabetes, median (IQR), months | 0 (0–0) | 60 (12–120) | 108 (48–168) | <0.001 |
| HbA1c, median (IQR), % | 5.0 (4.6–5.8) | 6.9 (6–8.5) | 7.5 (6.6–8.6) | 0.03 |
| FPG, median (IQR), mg/dL | 93.6 (86.4–106.2) | 134 (113.6–173.7) | 143.6 (104.9–188.3) | <0.001 |
| Hemoglobin, mean (SD), g/L | 131.8 (23.8) | 122 (20.3) | 129.5 (24.5) | 0.42 |
| Serum albumin, mean (SD), g/L | 37.3 (3.9) | 33.7 (5.7) | 36.6 (6.8) | 0.17 |
| eGFR, median (IQR), mL/min/1.73 m^2^ | 112.2 (117.3–124) | 101 (84–104.9) | 69.8 (49.4–89.8) | <0.001 |
| 24-h proteinuria, median (IQR), g/d | 0 (0–0) | 0 (0–0) | 2.8 (0.69–4.2) | <0.001 |

Data are presented as means (SDs) for continuous variables with a normal distribution, as medians (25^th^–75^th^ percentiles) for continuous variables without a normal distribution, and as percentages for categorical variables. BMI, body mass index; FPG, fasting plasma glucose; eGFR, estimated glomerular filtration rate; HbA1c, hemoglobin A1c.

**Supplementary Table S3. The clinical and pathological characteristics of participants enrolled in targeted proteomic study and participants who were not enrolled in the targeted proteomic study.**

| Characteristics | Cohort with eGFR decline trajectories (n=54) | Cohort without eGFR decline trajectories (n=89) | *p* value |
| --- | --- | --- | --- |
| Age, mean (SD), y | 52 (9) | 50 (10) | 0.41 |
| Sex, Male, n (%) | 32(59.3) | 62(69.7) | 0.20 |
| Smoking, Never/Ex/Current, (n) | 34/5/15 | 51/12/26 | 0.70 |
| BMI, mean (SD), kg/m^2^ | 25.1 (3.2) | 25.6 (3) | 0.41 |
| SBP, mean (SD), mmHg | 140 (24) | 145 (22) | 0.18 |
| DBP, mean (SD), mmHg | 82 (12) | 84 (13) | 0.06 |
| Duration of diabetes, median (IQR), months | 108 (48–168) | 94 (36–132) | 0.08 |
| HbA1c, median (IQR), % | 7.5 (6.6–8.6) | 7.1 (6.1–8.3) | 0.12 |
| FPG, median (IQR), mg/dL | 143.6 (104.9–188.3) | 125.5 (103.3–164) | 0.49 |
| Hemoglobin, mean (SD), g/L | 129.5 (24.5) | 127.1 (26) | 0.60 |
| Serum albumin, mean (SD), g/L | 38.6 (6.8) | 37.3 (6.9) | 0.06 |
| eGFR, median (IQR), mL/min/1.73 m^2^ | 71.8 (49.4–94) | 62.2 (47.8–94.8) | 0.94 |
| 24-h proteinuria, median (IQR), g/d | 2.5 (0.69–5.2) | 3.6 (1.2–5.24) | 0.27 |
| uACR, median (IQR), (mg/g) | 1179 (414–1715) | 1262 (455–2013) | 0.09 |
| RAAS inhibitors, No. (%) | 48(88.9) | 71(79.8) | 0.16 |
| OHA therapy, No. (%) | 30(55.6) | 44(49.4) | 0.48 |
| Insulin therapy, No. (%) | 37(68.5) | 57(64.0) | 0.59 |
| Statins, No. (%) | 36(66.7) | 48(53.9) | 0.13 |
| RPS classification^†^, n (%) |  |  | 0.45 |
| I | 7(13.0) | 4(4.5) |  |
| IIa | 12(22.2) | 26(29.2) |  |
| IIb | 9(16.7) | 14(15.7) |  |
| III | 21(38.9) | 37(41.6) |  |
| IV | 5(9.2) | 8(9.0) |  |
| IFTA^†^, n (%) |  |  | 0.75 |
| score 0 | 5(9.3) | 3(3.4) |  |
| score 1 | 29(53.7) | 42(47.2) |  |
| score 2 | 17(31.5) | 37(41.5) |  |
| score 3 | 3(5.5) | 7(7.9) |  |
| Interstitial inflammation^†^, n (%) | |  | 0.10 |
| score 0 | 0(.0) | 4(4.5) |  |
| score 1 | 45(83.3) | 62(69.7) |  |
| score 2 | 9(16.7) | 23(25.8) |  |
| Arteriosclerosis^†^, n (%) |  |  | 0.55 |
| score 0 | 6(11.1) | 8(9.0) |  |
| score 1 | 24(44.4) | 48(53.9) |  |
| score 2 | 24(44.4) | 33(37.1) |  |
| Arteriolar hyalinosis^†^, n (%) | |  | 0.40 |
| score 0 | 4(7.4) | 7(7.9) |  |
| score 1 | 19(35.2) | 22(24.7) |  |
| score 2 | 31(57.4) | 60(67.4) |  |

^†^ Defined by RPS DN classification. Data are presented as means (SDs) for continuous variables with a normal distribution, as medians (25^th^–75^th^ percentiles) for continuous variables without a normal distribution, and as percentages for categorical variables. BMI, body mass index; SBP, systolic blood pressure; DBP, diastolic blood pressure; FPG, fasting plasma glucose; eGFR, estimated glomerular filtration rate; HbA1c, hemoglobin A1c; UACR, urine albumin-to-creatinine ratio; RAAS, renin-angiotensin-aldosterone system; OHA, oral hypoglycemic agent; RPS, Renal Pathology Society; IFTA, interstitial fibrosis and tubular atrophy.

**Supplementary Table S4. The clinical characteristics of participants in healthy control group, diabetes without chronic kidney disease group, and diabetic nephropathy group in targeted proteomic study**

| Characteristics | HC (n=29) | DM (n=22) | DN (n=54) | *p* value |
| --- | --- | --- | --- | --- |
| Age, mean (SD), y | 51 (9) | 54 (8) | 52 (9) | 0.56 |
| Sex, Male, n (%) | 16(55.2) | 13(59.1) | 32(59.3) | 0.93 |
| Smoking, Never/Ex/Current, (n) | 16/5/8 | 10/6/6 | 34/5/15 | 0.36 |
| BMI, mean (SD), kg/m2 | 21.9 (3) | 23.7 (2.6) | 25.1 (3.2) | <0.001 |
| SBP, mean (SD), mmHg | 120 (17) | 136 (19) | 140 (24) | 0.01 |
| DBP, mean (SD), mmHg | 76 (10) | 81 (12) | 82 (12) | 0.08 |
| Duration of diabetes, median (IQR), months | 0 (0–0) | 60 (12–120) | 108 (48–168) | <0.001 |
| FPG, median (IQR), mg/dL | 93.6 (86.4–106.2) | 134 (113.6–173.7) | 143.6 (104.9–188.3) | <0.001 |
| Hemoglobin, mean (SD), g/L | 131.8 (23.8) | 122 (20.3) | 129.5 (24.5) | 0.31 |
| Serum albumin, mean (SD), g/L | 38 (3.4) | 34.1 (5.7) | 38.6 (6.8) | 0.1 |
| eGFR, median (IQR), mL/min/1.73 m2 | 119.2 (117.3–124) | 98 (84–104.9) | 71.8 (49.4–94) | <0.001 |
| 24-h proteinuria, median (IQR), g/d | 0 (0–0) | 0 (0–0) | 2.5 (0.69–5.2) | <0.001 |

Data are presented as means (SDs) for continuous variables with a normal distribution, as medians (25^th^–75^th^ percentiles) for continuous variables without a normal distribution, and as percentages for categorical variables. BMI, body mass index; SBP, systolic blood pressure; DBP, diastolic blood pressure; FPG, fasting plasma glucose; eGFR, estimated glomerular filtration rate; HC, healthy control; DM, diabetes; DN, diabetic nephropathy.

**Supplementary Table 5. Urinary complement proteins associated with a faster estimate glomerular filtration rate decline by linear regression analyses.**

|  | Univariate | |  | Multivariate ^a^ | | |  | Multivariate ^b^ | | |
| --- | --- | --- | --- | --- | --- | --- | --- | --- | --- | --- |
| Complement proteins | R | *p* value |  | Standardized Coefficient B | (95% CI) | *p* value |  | Standardized Coefficient B | (95% CI) | *p* value |
| C1QA | -0.18 | 0.2 |  | 0.03 | (-0.29, 0.35) | 0.86 |  |  |  |  |
| C1S | 0.05 | 0.72 |  | 0.33 | (-0.04, 0.62) | 0.13 |  |  |  |  |
| C2 | 0.3 | 0.03 |  | 0.56 | (-0.21, 0.87) | 0.06 |  |  |  |  |
| C3 | 0.38 | <0.01 |  | 0.41 | (0.1, 0.54) | 0.01 |  | 0.30 | (-0.14, 0.6) | 0.22 |
| C4A | 0.24 | 0.08 |  | 0.36 | (-0.09, 0.71) | 0.16 |  |  |  |  |
| C4B | 0.17 | 0.21 |  | 0.31 | (-0.02, 0.64) | 0.24 |  |  |  |  |
| C5 | -0.10 | 0.46 |  | 0.17 | (0.11, 0.33) | 0.01 |  | 0.28 | (-0.03, 0.49) | 0.10 |
| C6 | 0.06 | 0.67 |  | 0.23 | (-0.05, 0.4) | 0.13 |  |  |  |  |
| C7 | -0.04 | 0.75 |  | 0.12 | (-0.26, 0.62) | 0.41 |  |  |  |  |
| C8A | 0.06 | 0.65 |  | 0.01 | (-0.33, 0.35) | 0.95 |  |  |  |  |
| C9 | 0.33 | 0.02 |  | 0.4 | (0.05, 0.74) | 0.03 |  | -0.06 | (-0.56, 0.45) | 0.83 |
| CFAI | 0.02 | 0.87 |  | 0.08 | (-0.29, 0.47) | 0.64 |  |  |  |  |
| CFAB | 0.03 | 0.83 |  | 0.33 | (-0.02, 0.51) | 0.07 |  |  |  |  |
| CFAH | 0.27 | 0.04 |  | 0.3 | (0.02, 0.45) | 0.03 |  | 0.20 | (0.03, 0.34) | 0.03 |
| FHR1 | 0.1 | 0.46 |  | 0.27 | (-0.05, 0.46) | 0.12 |  |  |  |  |
| FHR2 | 0.12 | 0.4 |  | 0.16 | (-0.11, 0.31) | 0.33 |  |  |  |  |
| DAF | -0.51 | <0.001 |  | -0.55 | (-0.84, -0.18) | <0.01 |  | -0.47 | (-0.79, -0.06) | 0.02 |
| CD59 | -0.53 | <0.001 |  | -0.4 | (-0.58, -0.1) | 0.01 |  | -0.40 | (-0.55, -0.14) | <0.01 |
| CLUS | 0.11 | 0.43 |  | 0.22 | (-0.14, 0.83) | 0.16 |  |  |  |  |

The two multivariate regression models were adjusted for age, sex, baseline log2 (baseline eGFR) and log2 (baseline urinary protein concentration). ^a^ Each complement protein in the urine was included individually in the model; ^b^ Complement protein with *p*<0.05 in the multivariate regression model were included together in the multivariate regression model.


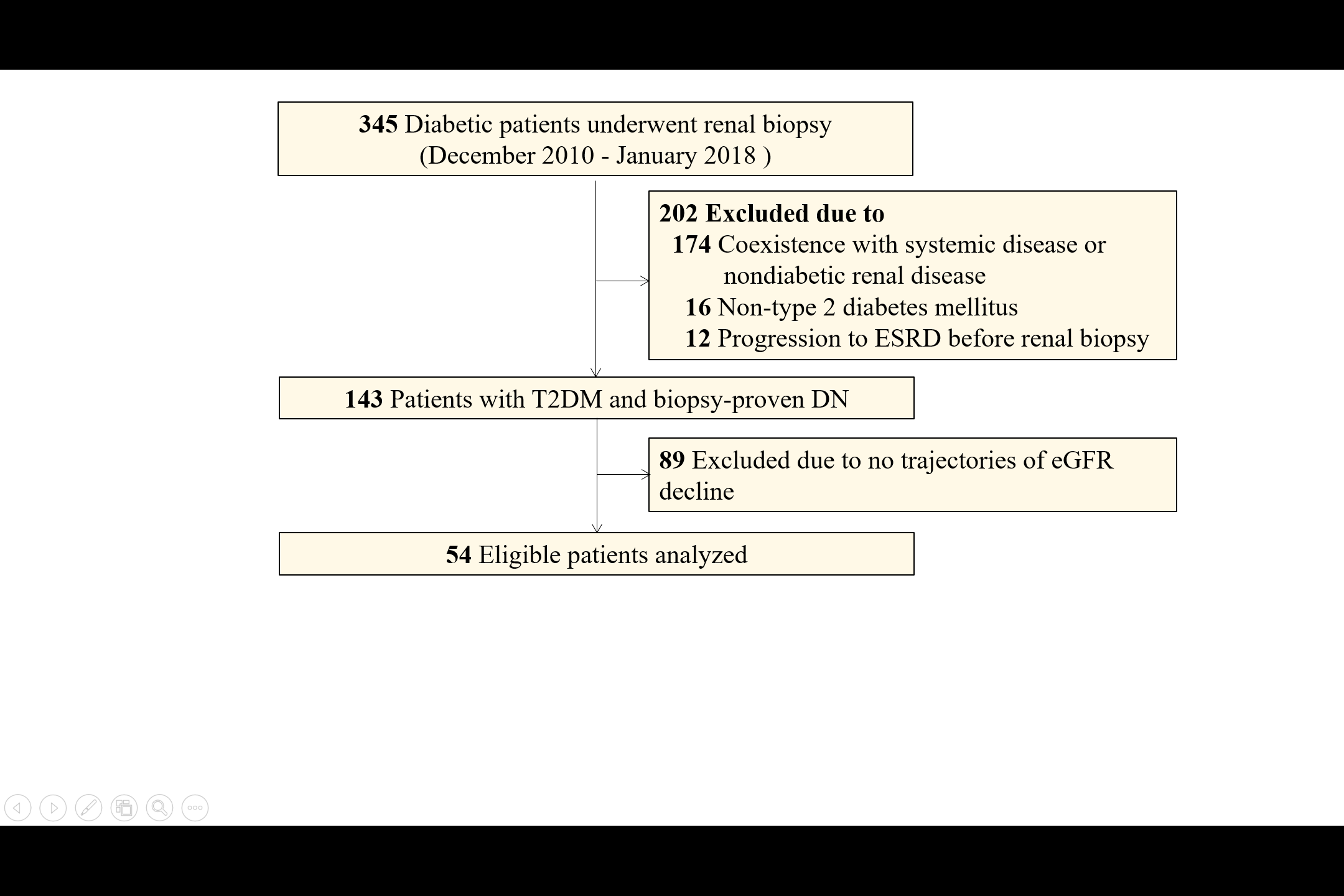


**Supplementary Figure 1. Flow chart of patients with type 2 diabetes and associated biopsy-proven diabetic nephropathy in this study. T2DM, type 2 diabetes mellitus; DN, diabetic nephropathy; ESRD, end-stage renal disease.**


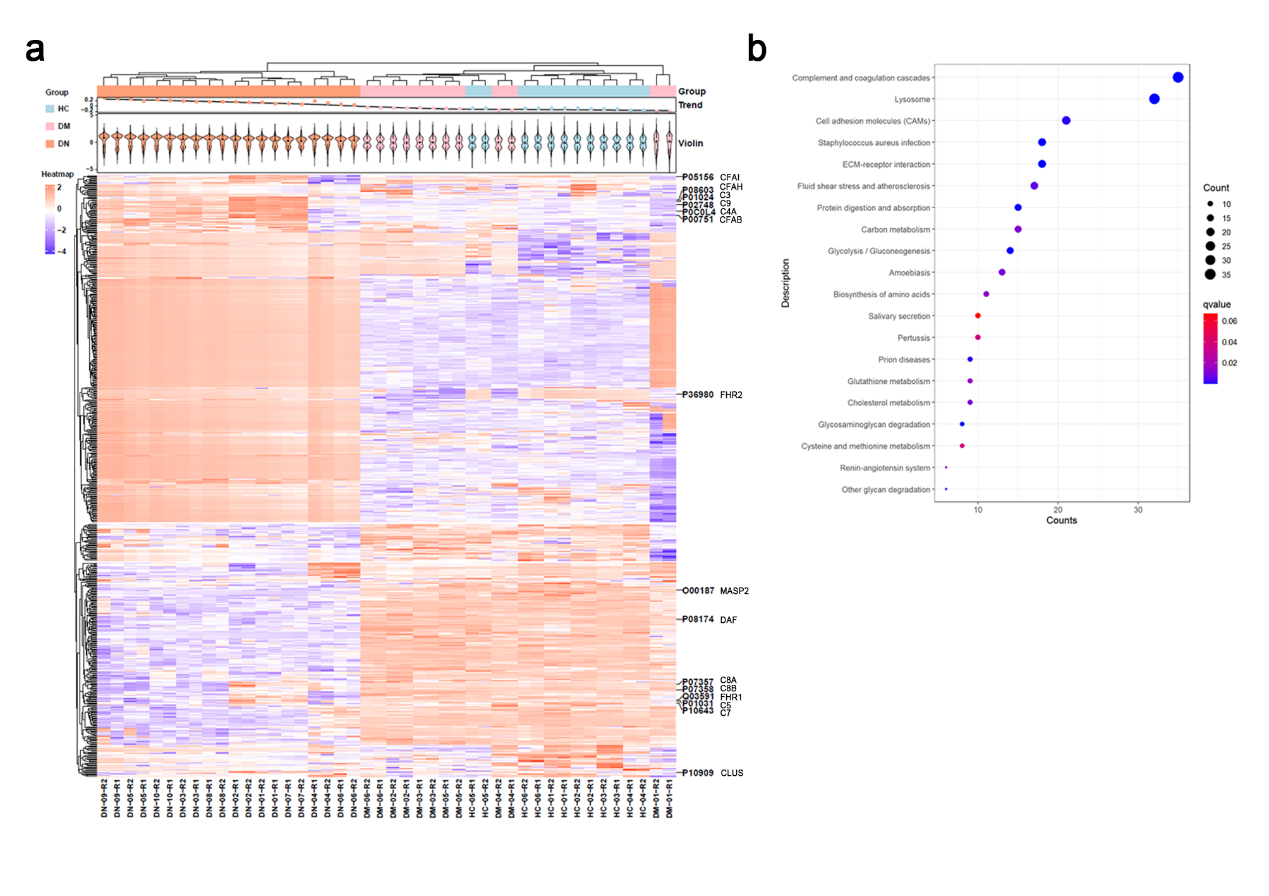


**Supplementary Figure 2. Urinary untargeted proteomic results by acetone precipitation method.** (a) Hierarchical cluster analysis of the upregulated and downregulated proteins in random urine from HC, DM, and DN groups. The red bar in the figure indicated the complement and complement regulatory proteins. (b) Functional analysis by KEGG showed that the different expressed urinary proteins in pathways are linked to the complement and coagulation cascades. HC, healthy control; DM, diabetes mellitus; DN, diabetic nephropathy.
